# Conditions of Confinement in U.S. Carceral Facilities During COVID-19: Individuals Speak—Incarcerated During the COVID-19 Epidemic

**DOI:** 10.1089/heq.2022.0017

**Published:** 2023-04-28

**Authors:** Nicole Cassarino, Harika Dabbara, Carla B. Monteiro, Arthur Bembury, Leslie Credle, Uma Grandhi, Ankita Patil, Samantha White, Monik C. Jiménez

**Affiliations:** ^1^ Tufts University School of Medicine, Boston, Massachusetts, USA.; ^2^ Brigham & Women's Hospital, Department of Medicine, Division of Women's Health, Boston, Massachusetts, USA.; ^3^ Boston University School of Medicine, Boston, Massachusetts, USA.; ^4^ Brigham Health Bridge Clinic, Boston, Massachusetts, USA.; ^5^ Cape Verdean Social Workers Association, Boston, Massachusetts, USA.; ^6^ Partakers Organization—College Behind Bars, Auburndale, Massachusetts, USA.; ^7^ National Council for Incarcerated and Formerly Incarcerated Women and Girls, Roxbury, Massachusetts, USA.; ^8^ Justice 4 Housing, Boston, Massachusetts, USA.; ^9^ Families for Justice as Healing, Boston, Massachusetts, USA.; ^10^ University of California Santa Cruz, Santa Cruz, California, USA.; ^11^ Duke University School of Medicine, Durham, North Carolina, USA.

**Keywords:** incarceration, COVID-19, community-based participatory research, inequities

## Abstract

**Objectives:**

We aimed to describe conditions of confinement among people incarcerated in the United States during the coronavirus disease 2019 (COVID-19) pandemic using a community-science data collection approach.

**Methods:**

We developed a web-based survey with community partners to collect information on confinement conditions (COVID-19 safety, basic needs, support). Formerly incarcerated adults released after March 1, 2020, or nonincarcerated adults in communication with an incarcerated person (proxy) were recruited through social media from July 25, 2020 to March 27, 2021. Descriptive statistics were estimated in aggregate and separately by proxy or formerly incarcerated status. Responses between proxy and formerly incarcerated respondents were compared using Chi-square or Fisher's exact tests based on α=0.05.

**Results:**

Of 378 responses, 94% were by proxy, and 76% reflected state prison conditions. Participants reported inability to physically distance (≥6 ft at all times; 92%), inadequate access to soap (89%), water (46%), toilet paper (49%), and showers (68%) for incarcerated people. Among those receiving prepandemic mental health care, 75% reported reduced care for incarcerated people. Responses were consistent between formerly incarcerated and proxy respondents, although responses by formerly incarcerated people were limited.

**Conclusions:**

Our findings suggest that a web-based community-science data collection approach through nonincarcerated community members is feasible; however, recruitment of recently released individuals may require additional resources. Our data obtained primarily through individuals in communication with an incarcerated person suggest COVID-19 safety and basic needs were not sufficiently addressed within some carceral settings in 2020–2021. The perspectives of incarcerated individuals should be leveraged in assessing crisis–response strategies.

## Introduction

Over 2 years after the initial global response to coronavirus disease 2019 (COVID-19), the U.S. sits a top two lists: the rate of incarceration and the rate of COVID-19 deaths.^1,2^ In 2020, COVID-19 case rates among individuals in U.S. federal and state Departments of Corrections (DOCs) were 4.8–5.5 times higher than the general population.^3–5^ Previous outbreaks of influenza,^6–8^ Legionnaires' disease,^9^ H1N1,^10,11^ and tuberculosis^12^ were harbingers of carceral facilities' vulnerability to SARS-CoV-2. Conditions, such as overcrowding, inadequate ventilation, poor sanitation, and difficulty accessing personal protective equipment (PPE), have created environments primed for outbreaks.

Additionally, prevalent chronic conditions (i.e., diabetes, hypertension, and asthma) place incarcerated populations at greater risk of COVID-19-related morbidity and mortality.^13^ Despite the waning of the pandemic in the general population, conditions of confinement experienced by incarcerated populations can provide important insight toward planning humane responses to inevitable future infectious disease transmission.

While physical distancing, mask wearing, handwashing, and testing were recommended mitigation strategies by the Centers of Disease Control and Prevention (CDC) for carceral facilities,^14^ data indicate that mitigation strategies reported by state-level DOCs were irregularly implemented and punitive.^15,16^ Additionally, incarcerated individuals have reported inadequate access to soap, water, and disinfectant supplies,^17^ and extensive periods of lockdowns (suspension of activities and confinement restricted to housing areas) or solitary confinement (housing with minimal to rare contact with others) to achieve physical distancing.^17–19^

While anecdotal evidence is compelling, robust quantitative data from the perspectives of incarcerated people during the pandemic are limited,^20–22^ of which only two were from the United States.^21,22^ Yet, access to basic needs, medical care, and lockdown procedures were not examined. The unique perspective of directly impacted people, which has historically been ignored, is essential to the evaluation of emergent crisis response within carceral facilities. While community advocates identified the need for timely and wide-spread data on confinement conditions, obtaining research agreements from all U.S. carceral systems to directly survey currently incarcerated people was deemed a barrier to these urgent data needs during a rapidly changing public health emergency.

Therefore, we partnered with community advocates to design and disseminate a web-based survey to collect detailed data on conditions of confinement and examine the feasibility of collecting data through outreach to community members such as loved ones, professional partners, and recently released individuals. Our methodological approach demonstrates how community members can contribute to epidemiological data collection efforts, the identification of salient health outcomes, and potential discordance between policy and implementation.

## Methods

### Instrument development

In May 2020, a Community Advisory Board (CAB) was convened to examine conditions experienced by individuals incarcerated during the pandemic. The CAB included formerly incarcerated advocates from local and national organizations, Families for Justice as Healing, Justice 4 Housing, the National Council of Incarcerated and Formerly Incarcerated Women and Girls, and the Partakers Organization. Each CAB member requested the inclusion of statements of commitment to their community and organizational mission (Supplementary Data S1). At study onset, there were no validated instruments to examine confinement conditions among incarcerated individuals during the pandemic; therefore, we developed a survey of COVID-19 safety, basic needs, support, and demographic factors. Questions were developed based on carceral system mitigation strategies, CDC guidelines,^14^ community-informed concerns,^23^ anecdotal evidence, and surveys targeting the general population.

The instrument was piloted among 12 individuals who had been incarcerated and released after March 1, 2020 or were in communication with an incarcerated individual. The survey included questions regarding completion time, accessibility, and appropriateness of survey content. In partnership, we reviewed the pilot findings, implemented changes, and planned survey dissemination.

The final instrument consisted of 42 questions, (37 multiple choice, 5 open-response; Supplementary Data S2), of which three were administered only to proxies and seven only to formerly incarcerated respondents (FIR). Survey data were collected anonymously using Research Electronic Data Capture and distributed using a snowball sampling method through Twitter, Facebook, and by e-mail to community partner organizations, medical and legal professionals, and local politicians. Proxy respondents were allowed to indicate whether they were an individual responding on the behalf of an incarcerated loved one, or a medical or legal professional.

### Study sample

The final study sample comprised 378 adults (≥18 years) who met the following criteria: (1) formerly incarcerated individual released after March 1, 2020 (beginning of the U.S. pandemic response; *n*=21), or (2) had been in contact with someone currently incarcerated (proxy; *n*=357). To maximize reported data, questions could be skipped. Proxies in contact with multiple incarcerated individuals were asked to submit a separate survey for each person, unless proxies were legal/medical professionals, who were prompted to report the number of people for whom the information was applicable.

### Statistical analyses

All primary analyses were descriptive and thus did not include statistical hypothesis testing. Frequencies and relative frequencies were calculated for each question based on available data for all respondents in aggregate, with a missing indicator for skipped questions. Geographic regions were defined according to U.S. Census Bureau classifications.^24^ Illustrative qualitative data were selected to provide context to quantitative findings.

Results were reported in aggregate and stratified by participant type (proxy/FIR). We assessed feasibility by examining internal validity, completed questionnaires, sample size, and missingness. The distribution of responses (excluding missing values) by proxies compared with FIRs for questions regarding COVID-19 safety, basic needs, and support were compared to assess internal validity. Individual results for FIRs should be carefully considered as the sample size is small.

Comparisons were conducted using two-sided Fisher's exact tests based on an α=0.05. We also conducted sensitivity analyses to examine differences over time and geography for variables with inconsistencies between proxies and FIRs. All analyses were conducted using Stata/IC (version 16.1; College Station, TX).

### Statement of ethics

This study was approved by the Institutional Review Board of the Mass General Brigham and deemed exempt because no personal identifiers were collected and the research posed minimal risk. All procedures were in accordance with institutional guidelines. However, we acknowledge that these data represent individuals who have been incarcerated and those who know them personally.

## Results

Of the 378 responses collected between July 28, 2020 and March 27, 2021, 94.4% (*n*=357) were proxies who completed the survey based on experiences shared by someone currently incarcerated (Table 1). The remaining 5.6% (*n*=21) were FIRs released after March 1, 2020. Most respondents provided data on facilities in the Northeast (37.0%), followed by the West (28.7%), South (26.1%), and Midwest (8.2%). In addition, 75.7% of respondents reflected conditions in state prisons, followed by jails (13.5%), federal prisons (5.8%), and other facilities, including immigration and customs enforcement (ICE) and juvenile detention centers (5.0%). Among respondents who reported gender (*n*=182) and race (*n*=163) of the impacted person, 39.6% were identified as female, 60.1% were White, 21.5% were Black, and 12.9% were Hispanic/Latinx.

**Table 1.** Facility and Demographic Information of Incarcerated People Represented

***N* = 378**	***n***	**%**
Role		
Proxy respondent	357	94.4
Formerly incarcerated	21	5.6
Age, years		
18–30	27	14.8
30–44	90	49.5
45–64	58	31.9
>65	7	3.9
Region of carceral facility^a^		
Southern U.S.	98	26.1
Northeastern U.S.	139	37.0
Western U.S.	108	28.7
Midwestern U.S.	31	8.2
Type of carceral facility		
Jail	51	13.5
State prison	286	75.7
Federal prison	22	5.8
ICE detention center	11	2.9
Other	8	2.1
Racial/ethnic background		
American Indian/Alaska Native	0	0.0
Asian	4	2.5
Black	35	21.5
Hispanic/Latinx	21	12.9
Native Hawaiian/Pacific Islander	1	0.6
White	98	60.1
Multiracial	4	2.5
Sex and gender		
Male	106	58.2
Female	72	39.6
Trans man	1	0.6
Trans woman	1	0.6
Nonbinary	2	1.1

All responses exclude “Don't know” and skipped questions.

^a^ Southern: Alabama, Arkansas, Delaware, DC, Florida, Georgia, Kentucky, Louisiana, Maryland, Mississippi, North Carolina, Oklahoma, South Carolina, Tennessee, Texas, Virginia, West Virginia.

Northeastern: Connecticut, Maine, Massachusetts, New Hampshire, New Jersey, New York, Pennsylvania, Rhode Island, Vermont.

Western: Alaska, Arizona, California, Colorado, Hawaii, Idaho, Montana, Nevada, New Mexico, Oregon, Utah, Washington, Wyoming.

Midwestern: Indiana, Illinois, Iowa, Kansas, Michigan, Minnesota, Missouri, Nebraska, North Dakota, Ohio, South Dakota, Wisconsin.

ICE, immigration and customs enforcement.

### COVID-19 safety

Responses about COVID-19 safety measures and access to basic needs are reported in aggregate and by respondent role (Table 2). Most participants reported exposure to lockdown procedures by incarcerated people (proxy=89.9%, FIR=79.0%). A length of confinement >20 h/day was reported by 86% of proxies and 93% of FIRs, with 50% of proxies and 33% of FIRs reporting a duration of >3 months. Over 90% of total respondents indicated an inability of incarcerated people to maintain the CDC-recommended six-foot distance from others, and nearly 30% of both groups reported individuals were housed with ≥2 people. Reports of PPE provision for incarcerated people (primarily face masks) were higher among proxies (83%) than FIRs (61%), whereas 85% and 79%, respectively, reported inconsistent staff use of PPE.

**Table 2.** Distribution of Reported Conditions of Confinement by Proxy and Formerly Incarcerated Respondents

	Overall		Proxy respondents		Formerly incarcerated respondents	
	***N***	%^a^	***n***	%^a^	***n***	%^a^
*N*	378	—	357	94.4	21	5.6
Lockdown						
Any lockdown due to COVID-19^b^	336	93.6	321	89.9	15	79.0
Duration of lockdown						
<2 weeks	17	5.8	17	6.2	0	0
>2 weeks to <1 month	51	17.5	48	17.4	3	20.0
1–2 months	47	16.2	43	15.6	4	26.7
2–3 months	34	11.7	31	11.2	3	20.0
>3 months	142	48.8	137	49.6	5	33.3
Hours per day on lockdown, hours/day						
≤20	35	13.4	34	13.8	1	6.7
>20	227	86.6	213	86.2	14	93.3
COVID-19 safety						
Unable to physically distance at all times	303	92.1	285	92.2	18	90.0
Number of people in cell						
Single	55	16.1	53	16.5	2	10.5
Double	183	53.7	171	53.1	12	63.2
>2/barracks/dormitory	103	30.2	98	30.4	5	26.3
Disinfection of common items						
No	147	58.6	139	59.4	8	47.1
More than daily	30	12.0	24	10.3	6	35.3
At least daily	74	29.5	71	30.3	3	17.7
Staff wearing PPE						
None	19	6.5	15	5.5	4	21.1
Some staff	230	78.8	219	80.2	11	57.9
All staff	43	14.7	39	14.3	4	21.1
Type of PPE worn by staff^b^						
Face mask	259	94.9	245	95.0	14	93.3
Gloves	79	28.9	71	27.5	8	53.3
Face shield	19	7.0	16	6.2	3	20.0
Incarcerated person given any PPE	244	81.6	233	82.9	11	61.1
Type of PPE received^b^						
Face Mask	240	98.4	230	98.7	10	90.9
Gloves	1	0.4	1	0.4	0	0
Homemade	6	2.5	5	2.2	1	9.1
Action taken if someone reported symptoms						
Isolation/quarantine	147	58.6	142	59.2	5	45.5
Returned to cell, IF cellmate ALSO has symptoms	15	6.0	15	6.3	0	0.0
Returned to cell, EVEN IF cellmate has *NO* symptoms	89	35.5	83	34.6	6	54.6
Able to reject COVID test without being punished	32	20.8	31	21.4	1	11.1
Type of information about COVID-19 received^b^						
No. of people with COVID in facility	124	32.8	124	34.7	0	0
No. of people tested for COVID in facility	87	23.0	87	24.4	0	0
No. of COVID deaths in facility	77	20.4	77	21.6	0	0
Plan for dealing with COVID	51	13.5	46	12.9	5	23.8
How to protect self	45	11.9	38	10.6	7	33.3
None	121	32.0	111	31.1	10	47.6
Basic needs						
Received free soap from the facility						
No	78	32.1	77	34.4	1	5.3
Yes, and enough for needs	28	11.5	23	10.3	5	26.3
Yes, but NOT enough for needs	137	56.42	124	55.4	13	68.4
Unable to access water when wanted	107	28.3	99	46.5	8	42.1
Unable to access enough toilet paper	96	48.5	89	49.7	7	36.8
Unable to shower every day if wanted	187	67.8	180	70.0	7	36.8
Delayed medical care for people with flu-like symptoms	191	81.3	177	81.2	14	82.4
Allowed to take possessions if moved within facility	87	46.5	83	48.3	4	26.7
Type of meals received						
1 hot meal/day and 2 bag lunches	120	62.5	107	60.8	13	81.3
Only bag lunches	72	37.5	69	39.2	3	18.8
Quantity of food received						
Enough	33	14.0	26	11.8	7	46.7
Not enough	203	86.0	195	88.2	8	53.3
Support						
Mental health care changes^c^						
More care received	7	4.6	6	4.1	1	11.1
Less care received	115	74.7	110	75.9	5	55.6
No care received	32	20.8	29	20.0	3	33.3
Given more stamps, telephone or video calls^b^	136	57.6	131	51.2	5	33.3
Able to use increased telephone or video calls						
Able to use for full allotted time	47	37.3	43	35.5	4	80.0
Able to use but not for full allotted time	52	41.3	52	43.0	0	0
Not able to use	27	21.4	26	21.5	1	20.0
Changes in receipt of legal aid						
More aid	5	2.6	5	2.8	0	0
Less aid	155	79.1	147	80.8	8	57.1
No change	36	18.4	30	16.5	6	42.9
Changes in parole hearings						
Paused/delayed	50	36.0	47	36.2	3	33.3
Limited access to parole hearings	38	27.3	37	28.5	1	11.1
Remote/full	12	8.6	11	8.5	1	11.1
No change	39	28.1	35	26.9	4	44.4

^a^ Excluding “don't know” and “other” answers.

^b^ Binary Y/N question. Yes was used as reference and reported. Responses are not mutually exclusive.

^c^ Changes in mental health care were collected among those who were currently receiving mental health care.

PPE, personal protective equipment.

Furthermore, over one-third of respondents overall reported that individuals who experienced COVID-19 symptoms were returned to their cells, irrespective of their cellmate's symptoms (proxy=35%, FIR=55%). Additionally, participants reported that individuals were unable to take their belongings if moved due to COVID-19 (proxy=5%, FIR=73%). One proxy respondent shared,

My husband caught covid because he was transferred from firecamp back to prison. Officially there were no transfers allowed during this time. He then got infected back at the main prison, got thrown into the SHU [Special Housing Unit]. He had none of his property, absolutely nothing for 2 weeks.

Lastly, >30% of respondents overall reported that facility-level information about COVID-19 was not shared (proxy=31%, FIR=48%).

### Basic needs, medical care, and support

Overall, responses indicated inadequate access to basic necessities among incarcerated people (Table 2). Although most indicated that incarcerated people received free soap (proxy=66%, FIR=95%), only a small proportion reported it was enough (proxy=10%, FIR=26%). Moreover, compared with prepandemic conditions, proxies and FIRs indicated less access to water (proxy=47%, FIRs=42%), toilet paper (proxy=50%, FIRs=37%), or daily showers (proxy=70%, FIRs=37%), and an insufficient food supply (proxy=88%, FIRs=53%).

Medical care and support resources were also affected by the pandemic response (Table 2). A delay beyond normal delivery of medical care for flu-like symptoms was reported by >80% of respondents overall (proxy=81%, FIR=82%). One proxy respondent reported,

My loved one recently got infected with COVID-19 and is also suffering for underlying medical conditions (chronic Hepatitis B and asthma). He didn't receive any medical treatments other than having his inhaler with him… The only “treatment” that was given to some individuals were over-the-counter medications such as Tylenol to reduce some flu-like symptoms. My loved one is still suffering from some lingering symptoms such as wheezing and coughing.

Similarly, of those receiving mental health care before the pandemic, 96% of proxies and 89% of FIRs reported less or no care received. Access to legal services were also reportedly reduced (proxy=81%, FIR=57%) and parole hearings delayed (>30% in both groups).

### Questions only answered by FIR

Questions about PPE replacement, increased stress, and receipt of financial support were asked only to FIRs (*n*=21; Table 3). Eighty-two percent indicated new PPE was provided less than once per week. Additionally, 88% reported concerns about contracting COVID-19 “most of the time” and experienced “a lot of added stress” (94%). Most FIRs (62.5%) also reported receiving less financial support during the study period and, only 17% reported receiving facility-level COVID-19 updates. One FIR shared,

**Table 3.** Questions Specifically for Formerly Incarcerated or Proxy Respondents

	***n***	%^a^
Formerly incarcerated respondents only (*n*=21)		
Month released		
March 2020–May 2020	10	47.6
June 2020–August 2020	8	38.1
September 2020–November 2020	3	14.3
Received COVID-19 facility updates	3	16.7
Frequency of PPE replacement		
Not daily but more than once per week	2	18.2
Less than once per week	9	81.8
Worried about getting COVID-19		
Most of the time	14	87.5
Sometimes/Not much/none	2	12.5
Added stress because of COVID-19		
A lot of added stress	15	93.8
Some stress/A little/no added stress	1	6.3
Decreased financial support^a^	10	62.5
Interested in getting a vaccine^a^	9	64.3
Questions for proxy respondents only (*n*=357)		
Last communication with incarcerated person		
Less than 1 week ago	303	84.9
Between 1–2 weeks ago	33	9.2
Between 2 weeks to 1 month ago	13	3.6
More than 1 month ago	8	2.2
Concerned about contracting COVID-19 from incarcerated individual if released	126	49.8
Concerned about getting COVID-19 if facility opened visitation	169	70.1
Which of the following would proxy need to feel safe from contracting COVID-19 from incarcerated individual?^b^		
A rapid COVID test within a week	86	24.1
Knowing that they had been tested in the past month	27	7.6
Place in your home where they could quarantine	52	14.6
Increased expenses to support incarcerated person	185	81.9

^a^ Yes was used as reference and reported.

^b^ Binary Y/N question. Yes was used as reference and reported. Responses are not mutually exclusive.

It felt as if the jail sacrificing me to get covid by locking me down with a person who had Covid. They didn't care if I got sick or not and made no effort to separate me from the covid case.

Another FIR shared,

Being locked in a cell for 23 hours and 40 minutes a day for weeks at a time (whenever there was a report of a positive case among staff/incarcerated citizens was extremely stressful). I often considered hurting other prisoners or the guards simply because I was angry. I am not a violent person.

### Questions only answered by proxy respondents

Questions about recency of communication, COVID-19 concerns, and support of incarcerated individuals were specifically asked of proxies. Most had communicated with the individual within the past week (85%) and reported increased expenses to support the individual (82%). One proxy respondent shared,

There is a food shortage and many are not opting for mess hall. I am supporting 10 women & it's difficult and expensive.

A high proportion (70%) reported concerns about contracting the virus upon visitation reinstatement or from the incarcerated individual if released (50%).

### Pilot feasibility

Overall, 23 surveys were initiated but not completed; however, response rates could not be calculated due to survey link distribution through social media and partner networks. Missingness was examined qualitatively to assess which variables were least likely to be answered by proxies or FIRs (Supplementary Data S3). Overall missing data for individual questions, which ranged from 5% for facility lockdown to 63% for changes to parole hearing, were higher for proxies than FIRs.

The distribution of responses between proxies and FIRs were compared for key metrics and were found to be consistent (*p*>0.05) for all but two questions (Table 4). Proxies were significantly more likely to report a facility being on lockdown (94%) than FIRs (79%, *p*=0.03) and fewer proxies (30%) reported that incarcerated individuals had access to daily showers compared with 63% of FIRs (*p*=0.01). However, cautious interpretation is warranted due to the sample size limitations.

**Table 4.** Examination of Internal Validity: Comparison of Proxy Respondents to Formerly Incarcerated Respondents

Question	Proxy respondents, % (***n***)	Formerly incarcerated respondents, % (***n***)	Two-sided Fisher's exact test ***p***-value
Facility on any type of lockdown	94.4 (320)	79.0 (15)	**0.03**
Hours spent in lockdown (>20 h/day)	86.2 (213)	93.3 (14)	0.70
Lockdown time period exceeding 3 months	49.6 (137)	33.3 (5)	0.29
Unable to maintain 6-foot distance at all times	92.3 (285)	90.0 (18)	0.67
Routine disinfection of commonly used surfaces	40.6 (95)	52.9 (9)	0.32
Adequate access to toilet paper	50.3 (90)	63.2 (12)	0.34
Ability to shower every day, if wanted	30.0 (77)	63.2 (12)	**0.01**
Adequate access to water	53.5 (114)	57.9 (11)	0.81
Enough free soap for needs	10.3 (23)	26.3 (5)	0.05
Isolated/Quarantined from cellmates if experiencing COVID-19 symptoms	59.2 (142)	45.5 (5)	0.37
Longer waits than normal to access medical care	81.2 (177)	82.4 (14)	1.00
Increased access to mental health care	4.1 (6)	11.1 (1)	0.35

Bolded values indicate a *p* < 0.05, corresponding to a statistically significant result.

All responses are dichotomized; values represent responses to “yes.”

## Discussion

To our knowledge, this is the first study designed and distributed in partnership with formerly incarcerated individuals to collect data on confinement conditions among individuals incarcerated across multiple states and systems. Our community-science data collection methods facilitated data transparency in a time-constrained setting by leveraging the strength of community ties. Our findings, primarily by proxy reports, suggest that in at least some facilities, individuals reported being unable to maintain physical distance, despite extensive lockdown periods, and lacked access to basic needs (i.e., soap, water, toilet paper, and showers). Respondents also indicated that incarcerated people had limited access to health care and legal aid. Lastly, the feasibility of this method was demonstrated by the ample sample size, and the comparability of findings to other sources of data (i.e., academic and popular press); however, resources would be needed to further engage formerly incarcerated individuals.

Understanding conditions of confinement during the COVID-19 pandemic from the perspective of incarcerated individuals is critical for crisis response planning and surveillance of standard operating procedures since public-facing policies may not align with actual implementation. Yet, incarcerated people have limited opportunities to challenge deviations from protocol.^25,26^ Moreover, carceral systems are generally not mandated to publicly share data on the health of incarcerated people, impairing external oversight.^27^ Unsurprisingly, there are limited data examining the experiences of the incarcerated during the pandemic.^20–22^ Among the few studies available,^20–22^ only two addressed confinement conditions in the United States.^21,22^

Between April and November 2020, the COVID-19 Questionnaire for Correctional Populations (CQCP) survey was administered to 327 individuals incarcerated in three U.S. states to collect data on mitigation strategies among staff and incarcerated individuals.^22^ Our findings were similar to the CQCP study for some conditions, such as soap provision (67.9% vs. CQCP=70.6%) but differed for physical distancing (7.9% vs. CQCP=66.4%). This may be due to wording differences, such as the CQCP asking participants about the ability to maintain physical distancing “if possible” rather than “at all times.” Our study expands our understanding by increasing geographic variability, reflecting a longer time period (July 2020–March 2021 vs. April 2020–November 2020), and by including questions on lockdown, basic needs, and mental health care access.

Furthermore, a mixed methods study was conducted to understand the perceived risk of COVID-19 among 41 incarcerated males experiencing ≥20 h/day of solitary confinement.^21^ In contrast to our findings, participants reported they felt more protected from COVID-19 than the general incarcerated population because of the solitary confinement measures they were subjected to, but reported infection was inevitable due to staff interactions and unsanitary food distribution. However, due to the small and highly selected sample, comparability of findings may be limited.

Our findings also paralleled data published by the popular media. The Essie Justice Group conducted a survey of 729 people in contact with an incarcerated individual. Sixty-two percent reported the incarcerated individual feared losing their lives,^28^ similar to 88% of FIRs in this study who worried about getting COVID-19 “most of the time.” Similarly, a survey among 50 individuals formerly detained by ICE reported that 80% were not able to maintain physical distance while eating, consistent with the 92% in our study who reported an inability to maintain physical distance.^17^

**FIG. 1.**
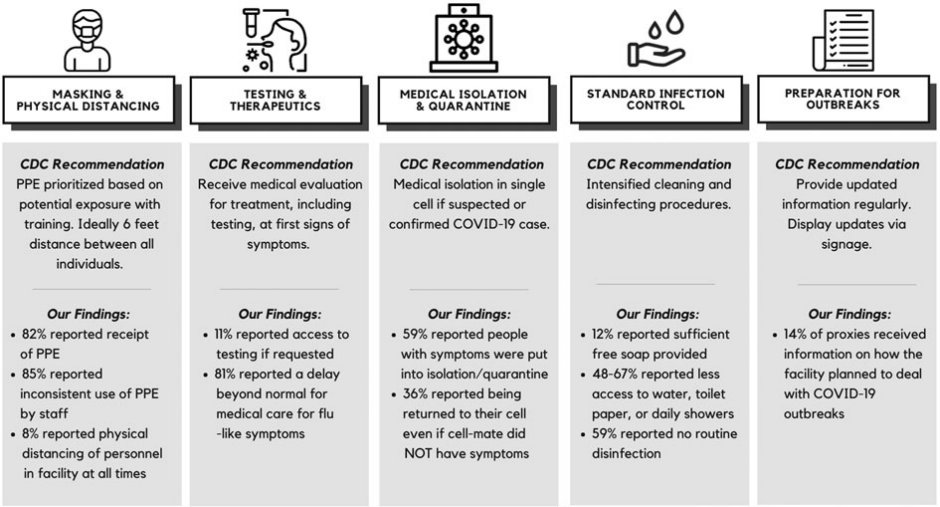
*Centers for Disease Control and Prevention. Section 3: Strategies for Everyday Operations vs. Enhanced COVID-19 Prevention Strategies. Guidance on Prevention and Management of Coronavirus Disease 2019 (COVID-19) in Correctional and Detention Facilities. 2022. Available from: https://www.cdc.gov/coronavirus/2019-ncov/community/correction-detention/guidance-correctional-detention.html#section_3; Vaccination is not presented given that the study was conducted before widespread vaccine availability in carceral facilities. CDC, Centers for Disease Control and Prevention; PPE, personal protective equipment.

Discrepancies between CDC recommended mitigation strategies and data reported in this study (Fig. 1) are further supported by emerging data. Based on data from the Bureau of Justice Statistics, wide variability in COVID-19 mitigation policies was observed across state and federal prisons. While all responding systems reported policies for the isolation or quarantine of symptomatic people in all facilities, only 8 systems reported testing staff in all facilities and only 13 tested incarcerated people in all facilities. Only 55% of systems reported giving PPE to incarcerated individuals, while others disallowed it or failed to mandate its use, aligning with the low proportion of staff PPE use identified in our study. However, the timing of policy implementation was not reported and data for jails and detention centers are not unavailable.^16^

Furthermore, some mitigation strategies may lead to physical and psychological distress. Restrictive housing has been associated with psychological harm,^29–31^ including anxiety, depression, aggression, self-harm, and increased mortality postrelease,^29,32^ although it should be noted that empirical data are sparse and differences in study outcomes and design features limit consistency and generalizability of findings.^33^ Despite recommendations against long-term restrictive housing (>15 days) by the United Nations,^34^ U.S. Department of Justice,^35^ National Commission on Correctional Health Care,^36^ lockdowns have been a prioritized mitigation approach; however, the long-term consequences of its use during the pandemic and delays in medical and mental health care are uncertain.

Several limitations of this study should be considered. Generalizability of our findings may be limited and biased toward those with internet access and social media users. Responses were not uniformly geographically distributed due to the nature of our community partner networks; thus, results were not stratified by geographic location due to sample size considerations. Generalizability of our sample by race and ethnicity is also uncertain due to missing data for these variables. Although our sample may overrepresent conditions in female facilities given our partner networks, there is no evidence to suggest that conditions would be improved for incarcerated males or individuals of color. Furthermore, individual results for FIRs should be considered carefully given the small sample size. Despite these limitations, general comparisons with other studies support the observed patterns.

Additionally, data from proxies may not be robust for all variables indicated by high proportions of missing data for some questions, such as parole hearings. For example, when comparing proxy to FIR responses, significantly more FIRs reported ability to shower daily if wanted, while more proxies reported lockdown conditions. However, 16% of FIRs who answered the questions regarding showering and lockdown were released before May 2020, before widespread response in all states, and were incarcerated in Florida, potentially reflecting variability in state responses. Although sensitivity analysis suggested high internal validity for other variables, these data should be interpreted cautiously due to sample size constraints.

Finally, although we collected data on the date of release, we did not collect the duration of incarceration. Our data reflect protocols implemented within the 1st year of the pandemic; however, policies varied substantially across and within systems, were implemented at different periods of time during the pandemic and recommendations for mitigation shifted.^15,29^ Thus, we could not assess the length of exposure to long-term lockdown or other COVID-19 mitigation strategies and findings may not reflect current conditions. Although we did not specifically exclude correctional staff, this group was not specifically engaged given recruitment through our community partner networks; however, their perspective is valuable and should be considered in future work.

## Health Equity Implications

Our data demonstrate that some incarcerated people reported experiencing inadequate implementation of safety protocols, difficulty accessing basic needs, and were subjected to long periods of restricted confinement. Further work is needed to confirm these findings in a representative sample. Our methods demonstrate the power of grassroots action to design a culturally relevant instrument and research study to collect descriptive, surveillance data of urgent community-identified needs. Data collected through these approaches can aid in addressing concerns of data transparency and oversight, and further support humane policy implementation, such as the use of decarceration, diversion programs, and compassionate release. Therefore, we urge public health leaders to engage with community organizations to develop meaningful research questions, leverage community strengths, and shape policy.

## Supplementary Material

Supplemental data

Supplemental data

Supplemental data

## Abbreviations Used

CAB: Community Advisory Board
CDC: Centers of Disease Control and Prevention
COVID-19: coronavirus disease 2019
CQCP: Questionnaire for Correctional Populations
DOCs: Departments of Corrections
FIR: formerly incarcerated respondents
ICE: immigration and customs enforcement
PPE: personal protective equipment

## References

[1] States of Incarceration: The Global Context 2021. Prison Policy Initiative, 2021. Available from: https://www.prisonpolicy.org/global/2021.html [Last accessed: November 21, 2021].

[2] WHO Coronavirus (COVID-19) Dashboard. 2021. Available from: https://covid19.who.int/ [Last accessed: February 10, 2022].

[3] Saloner B, Parish K, Ward JA, DiLaura G, Dolovich S. COVID-19 cases and deaths in federal and state prisons. JAMA 2020;324(6):602–603. 32639537 10.1001/jama.2020.12528 PMC7344796

[4] Jiménez MC, Cowger TL, Simon LE, Behn M, Cassarino N, Bassett MT. Epidemiology of COVID-19 among incarcerated individuals and staff in Massachusetts jails and prisons. JAMA Netw Open 2020;3:e2018851. 32821919 10.1001/jamanetworkopen.2020.18851 PMC7442924

[5] Hagan LM, Williams SP, Spaulding AC, et al. Mass testing for SARS-CoV-2 in 16 prisons and jails—Six jurisdictions, United States, April–May 2020. MMWR Morb Mortal Wkly Rep 2020;69:1139–1143. 32817597 10.15585/mmwr.mm6933a3 PMC7439979

[6] Robinson S, Smith P, Stephen D, Shubert J, Reed C, Manning S. Influenza outbreaks at two correctional facilities—Maine, March 2011. Morb Mortal Wkly Rep 2012;61:229–232. 22475851

[7] Besney J, Moreau D, Jacobs A, et al. Influenza outbreak in a Canadian correctional facility. J Infect Prev 2017;18:193–198. 28989527 10.1177/1757177416689725 PMC5496689

[8] Young LC, Dwyer DE, Harris M, et al. Summer outbreak of respiratory disease in an Australian prison due to an influenza A/Fujian/411/2002(H3N2)-like virus. Epidemiol Infect 2005;133:107–112. 15724717 10.1017/s0950268804003243 PMC2870228

[9] Lucas KD, Wheeler C, McLendon P, Leistikow BN, Mohle-Boetani JC. Outbreak of Legionnaires' disease associated with cooling towers at a California state prison, 2015. Epidemiol Infect 2018;146:297–302. 29386076 10.1017/S0950268818000110 PMC9134549

[10] Chao WC, Liu PY, Wu CL. Control of an H1N1 outbreak in a correctional facility in central Taiwan. J Microbiol Immunol Infect 2017;50:175–182. 26051221 10.1016/j.jmii.2015.05.005

[11] Turner KB, Levy MH. Prison outbreak: Pandemic (H1N1) 2009 in an Australian prison. Public Health 2010;124:119–121. 20149400 10.1016/j.puhe.2009.12.005

[12] Lambert LA, Armstrong LR, Lobato MN, Ho C, France AM, Haddad MB. Tuberculosis in jails and prisons: United States, 2002–2013. Am J Public Health 2016;106:2231–2237. 27631758 10.2105/AJPH.2016.303423 PMC5104991

[13] Nowotny KM, Rogers RG, Boardman JD. Racial disparities in health conditions among prisoners compared with the general population. SSM Popul Health 2017;3:487–496. 28824953 10.1016/j.ssmph.2017.05.011 PMC5558461

[14] COVID-19 and Your Health. U.S. Department of Health & Human Services 2022. Available from: https://www.cdc.gov/coronavirus/2019-ncov/your-health/need-to-know.html [Last accessed: February 20, 2022].

[15] Novisky MA, Narvey CS, Semenza DC. Institutional responses to the COVID-19 pandemic in American Prisons. Vict Offend 2020;15:1244–1261.

[16] Carson EA, Nadel M, Gaes G. Impact of COVID-19 on state and federal prisons, March 2020–February 2021. In: Bureau of Justice Statistics, ed. U.S. Department of Justice, Office of Justice Programs: Washington, DC, USA; 2022.

[17] Peeler K, Erfani P, Lee CH, et al. Praying for Hand Soap and Masks: Health and Human Rights Violations in U.S. Immigration Detention During the COVID-19 Pandemic. New York, NY, USA; 2021.

[18] What Happens When More Than 300,000 Prisoners Are Locked Down? The Marshall Project, 2020. Available from: https://www.themarshallproject.org/2020/04/15/what-happens-when-more-than-300-000-prisoners-are-locked-down [Last accessed: February 10, 2022].

[19] Association AB. ABA Standards for Criminal Justice: Treatment of Prisoners. Standard 23-10. American Bar Association: Washington, DC, USA; 2011.

[20] Di Giuseppe G, Pelullo CP, Lanzano R, Napolitano F, Pavia M. Knowledge, attitudes, and behavior of incarcerated people regarding COVID-19 and related vaccination: a survey in Italy. Sci Rep 2022;12:960. 35046470 10.1038/s41598-022-04919-3 PMC8770777

[21] Pyrooz DC, Labrecque RM, Tostlebe JJ, Useem B. Views on COVID-19 from Inside Prison: Perspectives of high-security prisoners. Just Eval J 2020;3:294–306.

[22] Pettus-Davis C, Kennedy SC, Veeh CA. Incarcerated individuals' experiences of COVID-19 in the United States. Int J Prison Health 2021;17(3):335–50. 10.1108/IJPH-11-2020-0094 33760428

[23] Berk J, Rich JD, Brinkley-Rubinstein L. What are the greatest health challenges facing people who are incarcerated? We need to ask them. Lancet Public Health 2021;6:e703–e704. 34115973 10.1016/S2468-2667(21)00074-8 PMC9814888

[24] 2010 Census Regions and Divisions of the United States. U.S. Census Bureau, 2010. Available from: https://www2.census.gov/geo/pdfs/maps-data/maps/reference/us_regdiv.pdf [Last accessed: February 10, 2022].

[25] Swearingen V. Negotiated governance in the prison inmate grievance process. Calif Law Rev 2008;96:1353–1382.

[26] Heffernan S. The way prisoners flag guard abuse, inadequate health care and unsanitary conditions is broken. ProPublica, December 2, 2020, 6 a.m. EST.

[27] Deitch M. But who oversees the overseers?: The status of prison and jail oversight in the United States. Am J Crim Law 2020;47:207–273.

[28] Clayton-Johnson G, Karefa-Johnson R, Rasaki T. Lives on the Line: Women with Incarcerated Loved Ones and the Impact of COVID-19 Behind Bars. Essie Justice Group: Oakland, CA, USA; 2020.

[29] Brinkley-Rubinstein L, Sivaraman J, Rosen DL, et al. Association of restrictive housing during incarceration with mortality after release. JAMA Netw Open 2019;2:e1912516. 31584680 10.1001/jamanetworkopen.2019.12516 PMC6784785

[30] Kaba F, Lewis A, Glowa-Kollisch S, et al. Solitary confinement and risk of self-harm among jail inmates. Am J Public Health 2014;104:442–447. 24521238 10.2105/AJPH.2013.301742 PMC3953781

[31] Haney C. Restricting the use of solitary confinement. Annu Rev Criminol 2018;1:285–310.

[32] Wildeman C, Andersen LH. Solitary confinement placement and post-release mortality risk among formerly incarcerated individuals: A population-based study. Lancet Public Health 2020;5:e107–e113. 32032555 10.1016/S2468-2667(19)30271-3

[33] Morgan RD, Gendreau P, Smith PM, et al. Quantitative syntheses of the effects of administrative segregation on inmates' well-being. Psychol Public Policy Law 2016;22:439–461.

[34] General Assembly of the United Nations. United Nations Standard Minimum Rules for the Treatment of Prisoners (the Nelson Mandela Rules). United Nations; 2016.

[35] U.S. Department of Justice. Report and Recommendations Concerning the Use of Restrictive Housing. In: Office of the Attorney General, ed. U.S. Department of Justice: Washington, DC, USA; 2016.

[36] Solitary Confinement (Isolation). National Commission on Correctional Health Care, 2016. Available from: https://www.ncchc.org/solitary-confinement-isolation-2016/ [Last accessed: June 16, 2022]. 10.1177/1078345816654233 27302711

