# Supplementary material for: Conditions of Confinement in U.S. Carceral Facilities During COVID-19: Individuals Speak—Incarcerated During the COVID-19 Epidemic
Source: Health Equity. 2023 Apr 28;7(1):261–70. doi: 10.1089/heq.2022.0017 (PMC10150723; doi:10.1089/heq.2022.0017)
Supplement: Supplemental data [file Suppl_MaterialS1.docx]

**Supplemental Material S1. Commitment Statement Community Advisory Board Members**

*“My work and experience with the criminal justice system has been the impetus that motivates me to seek solutions that can help reduce recidivism by providing essential resources to formerly* *incarcerated individuals ... I believe in working with the gatekeepers to ensure that funding is directed to critical areas that are needed most, and will have a significant impact on the population and the communities we work with.”* —Arthur Bembury

*“Because of the things I saw during my 19 years of incarceration I have become an advocate for the women I had to leave behind and the women that will sleep in a prison bed tomorrow.”*

—CAB Member

*“Surviving the Federal carceral punishment system is the motivation that drives the work I do today. At Justice 4 Housing we advocate for the abolishment of discriminatory housing policies and ending incarceration of women.”* —Leslie Credle
