# Supplementary material for: Conditions of Confinement in U.S. Carceral Facilities During COVID-19: Individuals Speak—Incarcerated During the COVID-19 Epidemic
Source: Health Equity. 2023 Apr 28;7(1):261–70. doi: 10.1089/heq.2022.0017 (PMC10150723; doi:10.1089/heq.2022.0017)
Supplement: Supplemental data [file Suppl_MaterialS3.docx]

**Supplemental Material S3. Distribution of Participant Responses with Missing Values**

|  | **Overall** | | **Proxy Respondents** | | **Formerly Incarcerated Respondents** | |
| --- | --- | --- | --- | --- | --- | --- |
|  | **N** | **%^a^** | **n** | **%^a^** | **n** | **%^a^** |
| **N** | 378 | - | 357 | 94.4 | 21 | 5.6 |
| **Lockdown** | | | | | | |
| **Any lock-down due to COVID-19^b^** | 378 |  | 357 |  | 21 |  |
| No | 22 | 5.8 | 18 | 5.0 | 4 | 19.1 |
| Yes | 337 | 89.2 | 322 | 90.2 | 15 | 71.4 |
| Missing | 19 | 5.0 | 17 | 4.8 | 2 | 9.5 |
|  |  |  |  |  |  |  |
| **Duration of lock-down*** | 337 |  | 322 |  | 15 |  |
| <2 weeks | 17 | 5.0 | 17 | 5.3 | - |  |
| >2 weeks - <1 month | 50 | 14.8 | 47 | 14.6 | 3 | 20.0 |
| 1-2 months | 47 | 14.0 | 43 | 13.4 | 4 | 26.7 |
| 2-3 months | 34 | 10.1 | 31 | 9.6 | 3 | 20.0 |
| > 3 months | 142 | 42.1 | 137 | 42.6 | 5 | 33.3 |
| Missing | 47 | 14.0 | 47 | 14.6 | - |  |
|  |  |  |  |  |  |  |
| **Hours per day on lock-down** | 337 |  | 322 |  | 15 |  |
| <20hrs/day | 35 | 10.4 | 34 | 10.6 | 1 | 6.7 |
| >20hrs/day | 227 | 67.4 | 213 | 66.2 | 14 | 93.3 |
| Missing | 75 | 22.3 | 75 | 23.3 | - |  |
|  |  |  |  |  |  |  |
| **COVID-19 Safety** | | | | | | |
| **Physical distancing at all times** | 378 |  | 357 |  | 2 |  |
| Yes | 26 | 6.9 | 24 | 6.7 | 2 | 9.5 |
| No | 303 | 80.2 | 285 | 79.8 | 18 | 85.7 |
| Missing | 49 | 13.0 | 48 | 13.5 | 1 | 4.8 |
|  |  |  |  |  |  |  |
| **Number of people in cell** | 378 |  | 357 |  | 21 |  |
| Single | 55 | 14.6 | 53 | 14.9 | 2 | 9.5 |
| Double | 183 | 48.4 | 171 | 47.9 | 12 | 57.1 |
| >2/barracks/dormitory | 103 | 27.3 | 98 | 27.5 | 5 | 23.8 |
| Missing | 37 | 9.8 | 35 | 9.8 | 2 | 9.5 |
|  |  |  |  |  |  |  |
| **Disinfection of common items** | 378 |  | 357 |  | 21 |  |
| No | 147 | 38.9 | 139 | 38.9 | 8 | 38.1 |
| More than daily | 30 | 7.9 | 24 | 6.7 | 6 | 28.6 |
| At least daily | 74 | 19.6 | 71 | 19.9 | 3 | 14.3 |
| Missing | 127 | 33.6 | 123 | 34.5 | 4 | 19.1 |
|  |  |  |  |  |  |  |
| **Staff wearing PPE** | 378 |  | 357 |  | 21 |  |
| None | 19 | 0.1 | 15 | 0.1 | 4 | 0.2 |
| Some staff | 230 | 0.8 | 219 | 0.8 | 11 | 0.6 |
| All staff | 43 | 0.1 | 39 | 0.1 | 4 | 0.2 |
| Missing | 86 | 22.8 | 84 | 23.5 | 2 | 9.5 |
|  |  |  |  |  |  |  |
| **Incarcerated Person Given any PPE** | 378 |  | 357 |  | 21 |  |
| No | 55 | 14.6 | 48 | 13.5 | 7 | 33.3 |
| Yes | 244 | 64.6 | 233 | 65.3 | 11 | 52.4 |
| Missing | 79 | 20.9 | 76 | 21.3 | 3 | 14.3 |
|  |  |  |  |  |  |  |
| **Action taken if someone reported symptoms** | 378 |  | 357 |  | 21 |  |
| Isolation/Quarantine | 147 | 38.9 | 142 | 39.8 | 5 | 23.8 |
| Returned to cell, IF cellmate ALSO has symptoms | 15 | 4.0 | 15 | 4.2 | - |  |
| Returned to cell, EVEN IF cellmate has *NO* symptoms | 89 | 23.5 | 83 | 23.3 | 6 | 0.5 |
| Missing | 127 | 33.6 | 117 | 32.8 | 10 | 47.6 |
|  |  |  |  |  |  |  |
| **Able to reject COVID test without being punished** | 378 |  | 357 |  | 21 |  |
| No | 122 | 32.3 | 114 | 31.9 | 8 | 38.1 |
| Yes | 32 | 8.5 | 31 | 8.7 | 1 | 4.8 |
| Missing | 224 | 59.3 | 212 | 59.4 | 12 | 57.1 |
|  |  |  |  |  |  |  |
| **Basic Needs** | | | | | | |
| **Received free soap from the facility** | 378 |  | 357 |  | 21 |  |
| No | 78 | 20.6 | 77 | 21.6 | 1 | 4.8 |
| Yes, and enough for needs | 28 | 7.4 | 23 | 6.4 | 5 | 23.8 |
| Yes, but NOT enough for needs | 137 | 36.2 | 124 | 34.7 | 13 | 61.9 |
| Missing | 135 | 35.7 | 133 | 37.3 | 2 | 9.5 |
|  |  |  |  |  |  |  |
| **Access water when wanted** | 378 |  | 357 |  | 21 |  |
| Yes | 125 | 33.1 | 114 | 31.9 | 11 | 52.4 |
| No | 107 | 28.3 | 99 | 27.7 | 8 | 38.1 |
| Missing | 146 | 38.6 | 144 | 40.3 | 2 | 9.5 |
|  |  |  |  |  |  |  |
| **Access enough toilet paper** | 378 |  | 357 |  | 21 |  |
| Yes | 102 | 27.0 | 90 | 25.2 | 12 | 57.1 |
| No | 96 | 25.4 | 89 | 24.9 | 7 | 33.3 |
| Missing | 180 | 47.6 | 178 | 49.9 | 2 | 9.5 |
|  |  |  |  |  |  |  |
| **Access to shower every day if wanted** | 378 |  | 357 |  | 21 |  |
| Yes | 89 | 23.5 | 77 | 21.6 | 12 | 57.1 |
| No | 187 | 49.5 | 180 | 50.4 | 7 | 33.3 |
| Missing | 102 | 27.0 | 100 | 28.0 | 2 | 9.5 |
|  |  |  |  |  |  |  |
| **Access to medical care for people with flu-like symptoms** | 378 |  | 357 |  | 21 |  |
| Delayed | 191 | 50.5 | 177 | 49.6 | 14 | 66.7 |
| Not Delayed | 44 | 11.6 | 41 | 11.5 | 3 | 14.3 |
| Missing | 143 | 37.8 | 139 | 38.9 | 4 | 19.1 |
|  |  |  |  |  |  |  |
| **Allowed to take possessions if moved within facility** | 378 |  | 357 |  | 21 |  |
| Yes | 87 | 23.0 | 83 | 23.3 | 4 | 19.1 |
| No | 100 | 26.5 | 89 | 24.9 | 11 | 52.4 |
| Missing | 191 | 50.5 | 185 | 51.8 | 6 | 28.6 |
|  |  |  |  |  |  |  |
| **Type of meals received** | 378 |  | 357 |  | 21 |  |
| 1 hot meal/day and 2 bag lunches | 120 | 31.8 | 107 | 30.0 | 13 | 61.9 |
| Only bag lunches | 72 | 19.1 | 69 | 19.3 | 3 | 14.3 |
| Missing | 186 | 49.2 | 181 | 50.7 | 5 | 23.8 |
|  |  |  |  |  |  |  |
| **Quantity of food received** | 378 |  | 357 |  | 21 |  |
| Enough | 33 | 8.7 | 26 | 7.3 | 7 | 33.3 |
| Not enough | 203 | 53.7 | 195 | 54.6 | 8 | 38.1 |
| Missing | 142 | 37.6 | 136 | 38.1 | 6 | 28.6 |
|  |  |  |  |  |  |  |
| **Support** | | | | | | |
| **Mental healthcare changes^c^** | 378 |  | 357 |  | 21 |  |
| More care received | 7 | 1.9 | 6 | 1.7 | 1 | 4.8 |
| Less care received | 115 | 30.4 | 110 | 30.8 | 5 | 23.8 |
| No care received | 32 | 8.5 | 29 | 8.1 | 3 | 14.3 |
| Missing | 224 | 59.3 | 212 | 59.4 | 12 | 57.1 |
|  |  |  |  |  |  |  |
| **Given more stamps, telephone, or video calls^b^** | 378 |  | 357 |  | 21 |  |
| Yes | 136 | 36.0 | 131 | 36.7 | 5 | 23.8 |
| No | 130 | 34.4 | 120 | 33.6 | 10 | 47.6 |
| Missing | 112 | 29.6 | 106 | 29.7 | 6 | 28.6 |
|  |  |  |  |  |  |  |
| **Able to use increased telephone or video calls** |  |  |  |  |  |  |
| Able to use for full allotted time | 47 | 34.6 | 43 | 32.8 | 4 | 80.0 |
| Able to use but not for full allotted time | 52 | 36.8 | 52 | 38.2 | - |  |
| Not able to use | 27 | 16.9 | 22 | 16.8 | 1 | 20.0 |
| Missing | 16 | 11.8 | 16 | 12.2 | - |  |
|  |  |  |  |  |  |  |
| **Changes in receipt of legal aid** | 378 |  | 357 |  | 21 |  |
| More aid | 5 | 1.3 | 5 | 1.4 | - |  |
| Less aid | 155 | 41.0 | 147 | 41.2 | 8 | 38.1 |
| No change | 36 | 9.5 | 30 | 8.4 | 6 | 28.6 |
| Missing | 182 | 48.2 | 175 | 49.0 | 7 | 33.3 |
|  |  |  |  |  |  |  |
| **Changes in parole hearings** | 378 |  | 357 |  | 21 |  |
| Paused/delayed | 50 | 13.2 | 47 | 13.2 | 3 | 14.3 |
| Limited access to parole hearings | 38 | 10.1 | 37 | 10.4 | 1 | 4.8 |
| Remote/full | 12 | 3.2 | 11 | 3.1 | 1 | 4.8 |
| No change | 39 | 10.3 | 35 | 9.8 | 4 | 19.1 |
| Missing | 239 | 63.2 | 227 | 63.6 | 12 | 57.1 |
